# Molecular Identification and Epidemiological Features of Human Adenoviruses Associated with Acute Respiratory Infections in Hospitalized Children in Southern China, 2012-2013

**DOI:** 10.1371/journal.pone.0155412

**Published:** 2016-05-12

**Authors:** Yi Chen, Fanghua Liu, Changbing Wang, Mingqi Zhao, Li Deng, Jiayu Zhong, Yingying Zhang, Jun Ye, Shuping Jing, Zetao Cheng, Yongxin Guan, Yi Ma, Yuanyuan Sun, Bing Zhu, Qiwei Zhang

**Affiliations:** 1 Central Laboratory, Guangzhou Women and Children’s Medical Center, Guangzhou Medical University, Guangzhou, Guangdong 510120, China; 2 Biosafety Level-3 Laboratory, School of Public Health, Southern Medical University (Guangdong Provincial Key Laboratory of Tropical Disease Research), Guangzhou, Guangdong 510515, China; 3 Department of Ophthalmology, Howe Laboratory, Massachusetts Eye and Ear Infirmary, Harvard Medical School, Boston, Massachusetts, 02114, United States of America; University of Hong Kong, HONG KONG

## Abstract

**Background:**

Acute respiratory infections (ARI) are the major worldwide health problem associated with high morbidity and mortality rates. Human adenovirus (HAdV) is one of the most common pathogens associated with viral ARI, and thus calls for specific diagnosis and better understanding of the epidemiology and clinical characteristics.

**Methods:**

Total 4,130 children with ARI requiring hospitalization from 2012 to 2013 were retrospectively studied. Throat swab specimens were collected from each patient. Fluorescence Quantitative PCR was performed to detect adenovirus as well as other common ARI-related pathogens. The seven HAdV hypervariable regions (HVRs) of the hexon gene from fifty-seven HAdVs-positive samples collected in the seasonal peaks were sequenced. Phylogenetic analysis of HVRs was also conducted to confirm the molecular types and genetic variation. In addition, epidemiological features and co-infection with other human respiratory pathogens were investigated and analyzed.

**Results:**

Of 4,130 hospitalized pediatric patients tested, the positive rates of respiratory syncytial virus (RSV), Mycoplasma pneumoniae (MP), and HAdV were 13.7%, 13.2%, and 12.0%, respectively. The HAdV positive patients accounted for 7.9%, 17.2%, 17.5% and 10.7% in age groups <1, 1–3, 3–6 and 6–14 years, respectively. Eighty-four HAdV positive children were co-infected with other respiratory pathogens (84/495, 17.0%). The most common co-infection pathogens with HAdV were MP (57.1%) and Human Bocavirus (HBoV) (16.7%). The majority of HAdV infected patients were totally recovered (96.9%, 480/495); However, four (0.8%) patients, who were previously healthy and at the age of 2 years or younger died of pneumonia. Seasonal peaks of HAdV infection occurred in the summer season of 2012 and 2013; the predominant HAdV type was HAdV-3 (70%), followed by HAdV-7 (28%). These epidemiological features were different from those in Northern China. The HAdV-55 was identified and reported for the first time in Guangzhou metropolitan area. Phylogenetic analysis indicated that all the HVR sequences of the hexon gene of HAdV-3 and -7 strains have high similarity within their individual types, and these strains were also similar to those circulating in China currently, indicating the conservation of hexon genes of both HAdV-3 and HAdV-7.

**Conclusions:**

Knowledge of the epidemiological features and molecular types of HAdV, a major pathogen of pediatric ARI, as well as other co-infected respiratory pathogens circulating in Guangzhou, southern China, is vital to predict and prevent future disease outbreaks in children. This study will certainly facilitate HAdV vaccine development and treatment of HAdV infections in children.

## Introduction

Adenoviruses are non-enveloped, double stranded DNA viruses that vary in size from 70 to 100 nm [1]. Up to now, at least 68 genotypes of human adenovirus (HAdV) have been identified [2] and classified into 7 species from A to G [3] based on serology, whole-genome sequencing, and phylogenetic analyses [4]. HAdVs can cause a wide range of illnesses, such as acute respiratory infections (ARIs), gastroenteritis, conjunctivitis, cystitis, and meningoencephalitis. They are usually responsible for 5–7% of respiratory illnesses in infants and children [5]. ARI is one of the most common causes of morbidity and mortality in children. HAdV-3, -4, and -7 are the major HAdV types associated with ARI in children and adults in the world [6–14]. The re-emergent genotype HAdV-55 was recently reported in children with ARIs in Beijing and Shaanxi Province, China [15]. However, information on the epidemiological and clinical features of HAdV circulating in hospitalized children is limited in China.

The purpose of this retrospective study was to determine the prevalence, epidemiology as well as the types of HAdVs circulating among hospitalized children with ARI in Guangzhou, Southern China during 2012–2013. All the specimens were collected in Guangzhou Women and Children’s Medical Center (GWCMC), which has 1,358 beds, receives over 3,000,000 pediatric outpatient person-times and admits 57,000 inpatients each year from Guangzhou as well as other cities in Southern China.

In addition, a previous investigation found that some patients infected by HAdVs were also co-infected with other viral pathogens [16], leading to severe clinical consequences in hospitalized patients. Thus, co-infections with other respiratory viruses were also investigated in this study.

## Materials and Methods

### Patients and clinical specimens

From January 1, 2012 to December 31, 2013, 4,130 hospitalized pediatric patients (younger than 14 years old) with symptoms of ARI (at least two of the following symptoms: cough, pharyngeal discomfort, nasal obstruction, snivel, sneeze, sore throat, dyspnea, and fever) or diagnosed as pneumonia as assessed by means of chest radiography, were included in this study at GWCMC. Chest radiography was conducted according to the clinical situation of the patients, and pneumonia was classified as an acute illness with ICD-10 (international classification of diseases-10). The Patients with the following conditions were excluded from our study: HIV infection; leukemia; receiving immunosuppressive agents; chemotherapy; known or suspected active tuberculosis.

Clinical characteristics of the patients were retrospectively analyzed. Throat swabs were collected in 2.5 ml of viral transport medium and were delivered to the Central Laboratory of GWCMC, which was used for further respiratory pathogen detection. This project was approved by the Ethics Committee of the GWCMC and was carried out in accordance with the principles expressed in the Declaration of Helsinki. Data records and collected clinical specimens are de-identified and completely anonymous.

### Detection of adenovirus and other common respiratory pathogens with real-time PCR

Total nucleic acid was extracted from respiratory specimens using a QIAamp DNA Mini Kit (QIAgen), in accordance with the manufacturer’s protocol. Adenovirus and other ten common respiratory pathogens were detected using Taqman real-time PCR kit according to the manufacturer’s protocol, including influenza A virus (infA), influenza B virus (infB), parainfluenza virus (PIV), respiratory syncytial virus (RSV), enterovirus (EV), human metapneumovirus (hMPV), bocavirus (BOV), rhinovirus (RHV), Mycoplasma pneumoniae (MP), and Chlamydia pneumonia (CP) (Guangzhou HuYanSuo Medical Technology Co. LTD; China)[17–18].

### Sequencing, molecular typing and phylogenetic analysis of the hypervariable regions of HAdV hexon gene

The seven hypervariable regions (HVRs) of HAdV hexon gene [19–20] from positive specimens at the seasonal peak were PCR amplified, purified and directly sequenced. The HVRs of the hexon gene are responsible for the viral serum neutralization and contain serotype-specific residues [19–20]. Primers HVRF and HVRR were used in the PCR reaction to amplify the 1.6-kbp fragment of the seven HVRs, as described earlier [6]. The amplicon was submitted to Invitrogen (Guangzhou) for sequencing. Additional sequencing primer was also used (Hex1F: 5’-GCCAGAGCCTCAAGTTGGA-3’). The sequencing reaction was carried out by using an ABI Prism BigDye Terminator v3.1 Cycle Sequencing Ready Reaction kit with AmpliTaq DNA polymerase on an ABI 3730 DNA sequencer (Applied Biosystems). DNA sequencing reads were assembled into a single contig using the SEQMAN software from the Lasergene package (DNAStar; Madison, WI).

Molecular Evolutionary Genetics Analysis (MEGA) version 5.1.0 was used for phylogenetic analyses of HAdV HVRs [21–22]. Neighbor-joining trees with 1,000 boot-strap replicates were constructed using a maximum-composite-likelihood method with default parameters. Bootstrap numbers shown at the nodes indicate the percentages of 1,000 replications producing the clade, with values above 80 considered robust. Additional phylogenetic analyses were performed by the construction of maximum likelihood trees using the same MEGA 5.1.0. A total of 13 HVR sequences from prototype and circulating strains of HAdV-B in China were used for phylogenetic analysis. These are as follows (for reference, the names include the corresponding GenBank accession number, type, and strain name): AY599834_HAdV-3_GB, DQ099432_HAdV-3_Guangzhou01, AY594253_HAdV-4_RI-67, AY594255_HAdV-7_Gomen, KC440171_HAdV-7_DG01, AY163756_HAdV-11_Slobitski, AY803294_HAdV-14_de Wit, AY601636_HAdV-16_ch.79, AY601633_HAdV-21_AV-1645, AY737797_HAdV-34_Compton, AY128640_HAdV-35_Holden, AY737798_HAdV-50_Wan, and JX491639_HAdV-55_BJ01.

### Statistical analysis

The data were evaluated for statistical significance with Chi-square test and Fisher’s exact test where appropriate. All tests were two-tailed and the value of p<0.05 was considered to represent a statistically significant difference.

## Results

### Demographic data of the children admitted with acute respiratory infections

Total 4,130 samples were collected and analyzed from 2012 to 2013. It showed that 80.8% of the ARI pediatric patients were at age groups 0–1 and 1–3 years (Table 1). The HAdV-positive patients accounted for 7.9%, 17.2%, 17.5%, and 10.7% for age groups <1, 1–3, 3–6, and 6–14 years, respectively (Table 1). Although patients at age groups 1–3 and 3–6 had higher HAdV infection rates, but there was no statistical significance difference among different age groups. The total HAdV-positive rate is 12.0% (495/4,130) for the whole age group younger than 14. The male and female HAdV-positive rates were 11.9% and 12.0%, respectively. No significant gender difference was found (*p* = 0.793).

**Table 1:** Demographic data of all the children admitted with acute respiratory infections.

Variable	Sample tested(%)	HAdV-positive (%)
**Age (years)**		
0–1	2,170 (52.5)	172 (7.9)
1–3	1,168 (28.3)	201 (17.2)
3–6	550 (13.3)	96 (17.5)
6–14	242 (5.9)	26 (10.7)
Total	4,130 (100)	495 (12.0)
**Gender**		
Male	2,916	347 (11.9)
Female	1,214	148 (12.0)
*p*		0.793

### Respiratory pathogens detected in hospitalized patients during 2012–2013

Of 4,130 clinical samples collected from 2012 to 2013, RSV was the most prevalent pathogen, accounting for 13.7% (566 cases) (Table 2); the second common pathogen was MP (543 cases, 13.2%). Four hundred and ninety five HAdV-positive samples were also confirmed (12%). InfA was the fourth common pathogen (344 cases, 8.3%). The other respiratory pathogens, such as influenza B virus, parainfluenza virus, EV, HMPV, HBoV, RHV, and CP were also detected.

**Table 2:** HAdV co-infections with common respiratory pathogens.

pathogens	HAdV	CP	MP	EV	infA	infB	HBOV	PIV	RHV	RSV	HMPV
**Positive rate (%)**	12.0	1.1	13.2	2.5	8.3	3.8	3.3	2.5	5.0	13.7	2.9
**co-infection case with HAdV**	-	0	48	1	6	2	14	0	5	3	5

infA: influenza A virus, infB: influenza B virus, PIV: parainfluenza virus, RSV: respiratory syncytial virus, EV: enterovirus, HMPV: human metapneumovirus, HBoV: human bocavirus, RHV: rhinovirus, MP: mycoplasma pneumonia and CP: chlamydia pneumonia.

Of 495 HAdV-positive patients, 84 were co-infected with other respiratory pathogens (84/495, 17.0%). The most common co-infection pathogens with HAdV were MP and HBoV (48/84, 57.1%; 14/84, 16.7%, respectively). There were no HAdV-positive patients co-infected with CP or HPIV.

### Epidemiology of adenovirus during 2012–2013

**Fig 1:**
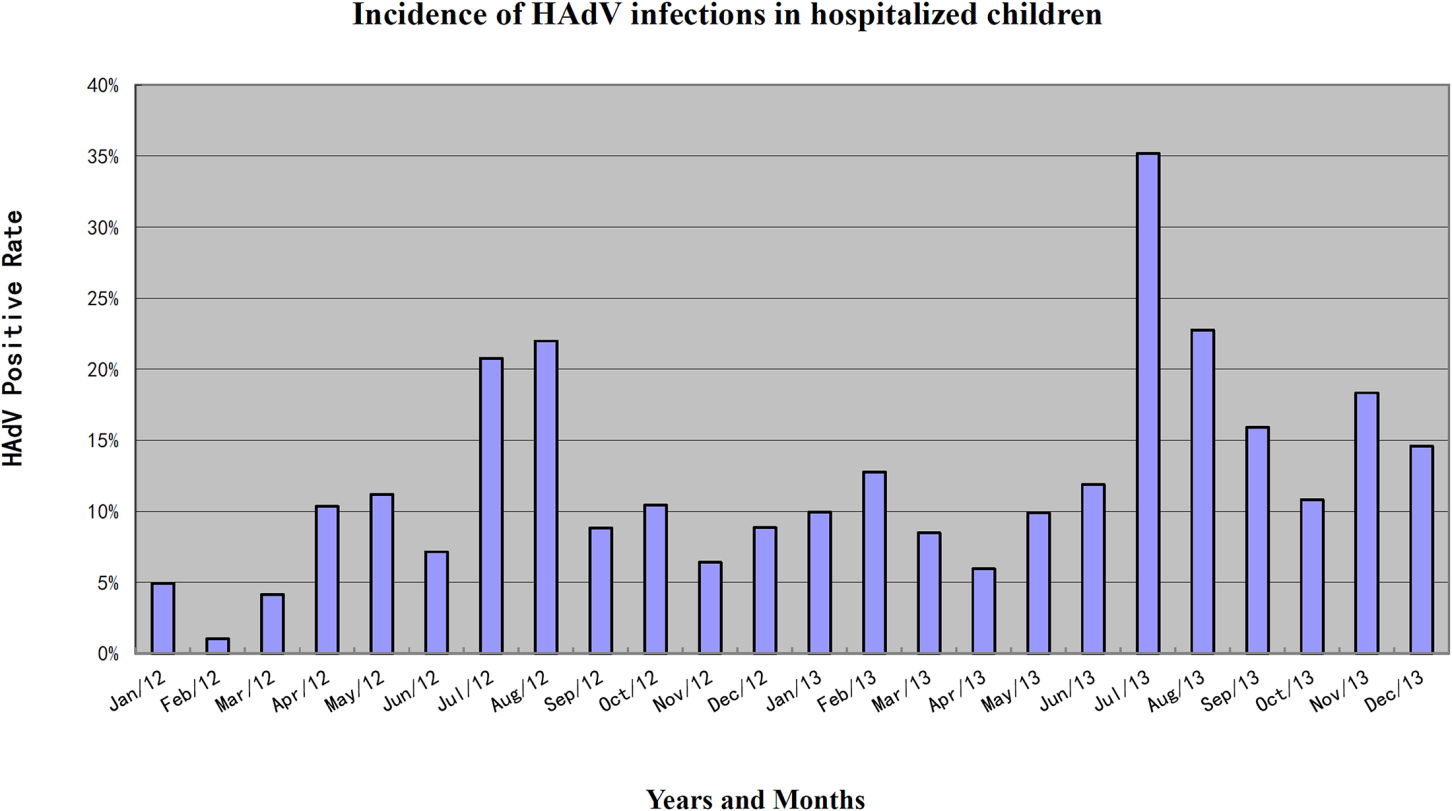
Monthly distribution of HAdV-positive hospitalized children.

During our 24-months study period, more than 100 respiratory samples each month were collected for respiratory pathogen detection. The HAdV-positive rate in patients admitted with ARI ranged from 1% to 35% each month (Fig 1; S1 Table). Low HAdV-positive rates were detected in February 2012 (2/195, 1.0%) and March 2012 (7/169, 4.1%), while unexpected high positive rates were found in July (57/162, 35.2%), August (43/189, 22.8%) of 2013, and July (27/130, 20.8%), August (31/141, 22.0%) of 2012, respectively.

The seasonal distributions of the HAdV infection from January 2012 to December 2013 were further analyzed (Table 3). HAdV was detectable throughout the year in pediatric patients. The highest HAdV-positive rate was found in summer (195 cases, 21.67%), which was significantly higher than the other seasons, including spring (9.44%), autumn (11.67%), and winter(6.44%) (p<0.05).

**Table 3:** Seasonal distribution of HAdV-positive cases from hospitalized children associated with acute respiratory infections.

	2012	2013
Winter (January-March)	17 (3.23%)	44 (10.48%)
Spring (April-June)	46 (9.79%)	70 (9.22%)
Summer (July-September)	67 (17.96%)*	128 (24.29%)*
Autumn (October-December)	42 (8.45%)	81 (14.54%)
*p* (χ^2^)	p<0.001* (χ^2^ = 64.006)	p<0.001* (χ^2^ = 57.257)

*A significant difference between summer and the other seasons.

The 495 HAdV-positive patients with ARI included 347 male and 148 female, and the median age was 21 months, ranging from 1 day to 13 years old. The clinical diagnoses included pneumonia, bronchitis, acute asthmatic bronchitis, and Tonsillitis (Table 4). The most common symptoms were fever (98.2%), cough (97.4%), and Dyspnea (72.1%); 208 patients (42%) had moist rales. Radiographic evidence of pneumonia was defined as the presence of consolidation (10.1%), alveolar or interstitial infiltrate (82.2%), and pleural effusion (1.8%). Thirty-two children (6.5%) diagnosed as pneumonia and with unstable vital signs were admitted into the Intensive Care Unit (ICU), 24 of which required respiratory support with mechanical ventilation. The ratio of male to female of these patients was 3:1, and the median age is 11 months, ranging from 54 days to 12 years old. Four hundred eighty HAdV-infected patients were totally recovered (96.9%,). However, four (0.8%) patients, who were previously healthy and aged 2 years and 1 month, 1 year and 3 months, 1 year and 1 month, and 8 months, respectively, died. Three of them were tested HAdV-positive only and the other one was co-infected with HBoV. They all suffered from high fever (>40°C) and rapid exacerbation of lower respiratory tract infection. All four cases required mechanical ventilation. Two of them died due to respiratory failure, and the other two died due to multiple organs dysfunction syndrome (MODS) and shock.

**Table 4:** Clinical characteristics of HAdV-positive hospitalized children.

Characteristics	HAdV positive (%) (N = 495)
**Symptom**	
Fever or feverish feeling	486 (98.2)
Cough	482 (97.4)
Dyspnea	357 (72.1)
Moist rales	208 (42.0)
**Radiographic finding**	
Consolidation	50 (10.1)
Alveolar or interstitial infiltrate	407 (82.2)
Pleural effusion	9 (1.8)
**Therapy**	
Intensive care	32 (6.5)
Mechanical Ventilation	24 (4.8)
**Prognosis**	
Totally recovery	480 (96.9)
Death	4 (0.8)

### Molecular types of HAdVs detected in hospitalized children with ARI in summers of 2012–2013

**Fig 2:**
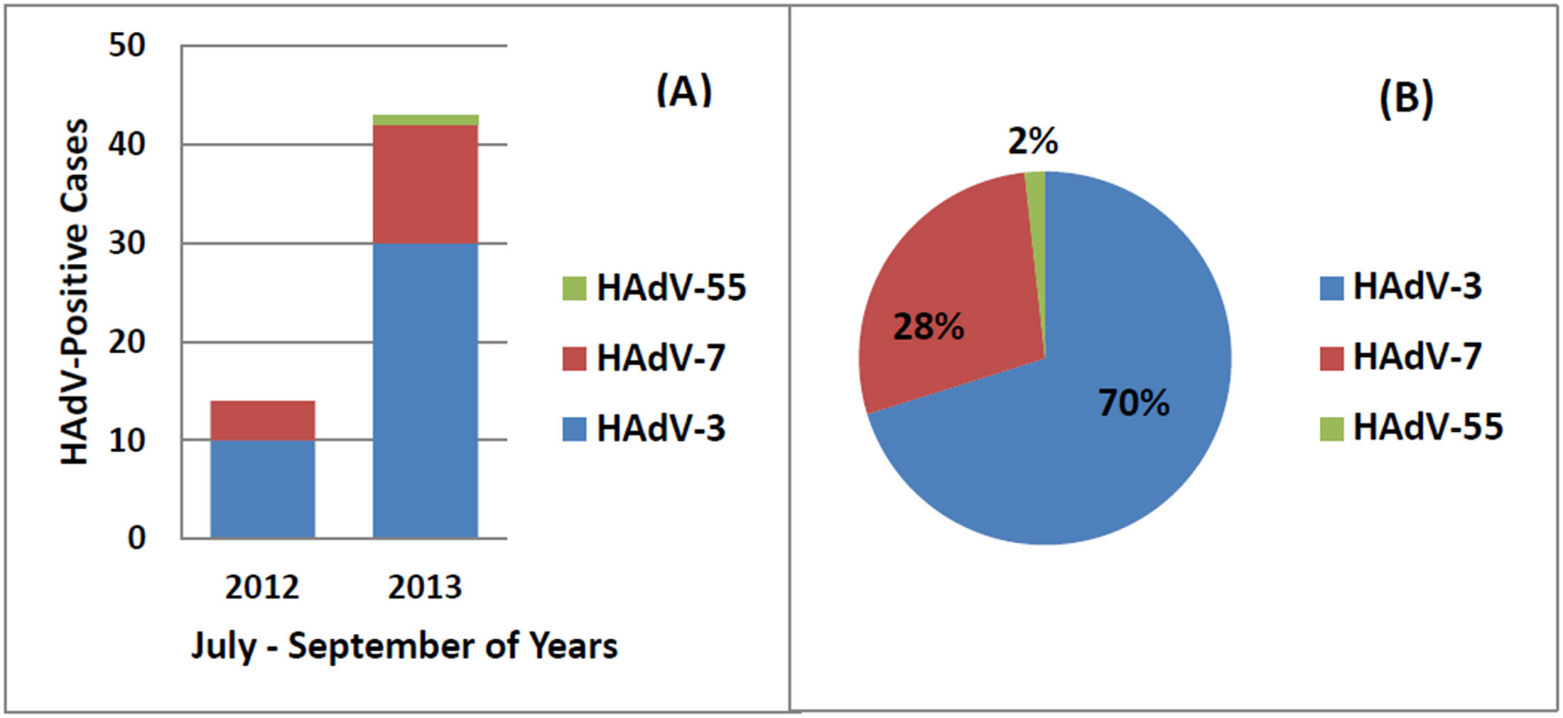
HAdV type distribution in hospitalized children with ARIs from July through September, 2012–2013. (A) HAdV type distribution from July through September in each year; (B) HAdV type distribution from July through September for the two years.

The 195 HAdV-positive samples during July to Spetember of 2012–2013 were collected. The viral DNA were extracted and the HVRs of HAdV hexon gene were amplified by PCR. Among these specimens, 57 were both amplified and sequenced successfuly for HVRs. The molecular types of these samples were determined by the Blastn of the sequences in GenBank. In 2012, 71.4% were identified as type 3 and 28.6% were type 7. In 2013, three types of HAdVs were identified: HAdV-3, -7 and -55. HAdV-3 account for 69.8% (30/43), followed by HAdV-7 (12, 27.9%) (Fig 2A). One case of HAdV-55 in 2013 was identified and reported for the first time in Guangzhou metropolitan area, after the first and second reports of this type in Shaanxi Province (2006) and Beijing (2012), China [15, 23] (Fig 2A). In summers of 2012–2013, the most prevalent HAdV type was HAdV-3, which accounted for 70% of the total cases (40/57); HAdV-7 accounted for 28% (16/57) (Fig 2B). No significant difference was found in clinical manifestations and laboratory findings when compared between the two HAdV types in these 56 cases (Table 5).

**Table 5:** Clinical manifestations and laboratory findings from hospitalized children infected with HAdV-3 and HAdV-7 in seasonal peaks, 2012–2013.

Characteristic	HAdV-3 (n = 40)	HAdV-7 (n = 16)	*p*
Age (years)			
0–3	33 (82.5)	11 (68.8)	0.257*
>3	7 (17.5)	5 (31.2)	
Sex (male, %)	29 (72.5)	12 (75.0)	0.849
Underlying diseases (%)	9 (22.5)	3 (18.8)	0.757
**Symptom and signs**			
Cough	38 (95.0)	15 (93.8)	0.851
Dyspnea	30 (75.0)	14 (87.5)	0.303
Diarrhea	5 (12.5)	3 (18.8)	0.546
Moist rale	21 (52.5)	8 (50.0)	0.866
**Laboratory tests**			
White blood cell (109/L)	9.0±3.9	7.6±3.1	0.201
Neutrophils (%)	49.8±16.2	40.3±20.7	0.074
Lymphocyte (%)	41.6±15.1	47.3±18.5	0.240
HGB (g/L)	107±14.5	111±14.7	0.325
PLT (10^9^/L)	345±123	269±86	0.029
**Outcome**			
Severe pneumonia	7 (17.5)	4 (25.0)	0.523

* *p* value between the HAdV-3 and HAdV-7-positive groups of all ages.

### Phylogenetic analysis of the hexon genes of HAdVs from hospitalized cases in Guangzhou, Southern China

**Fig 3:**
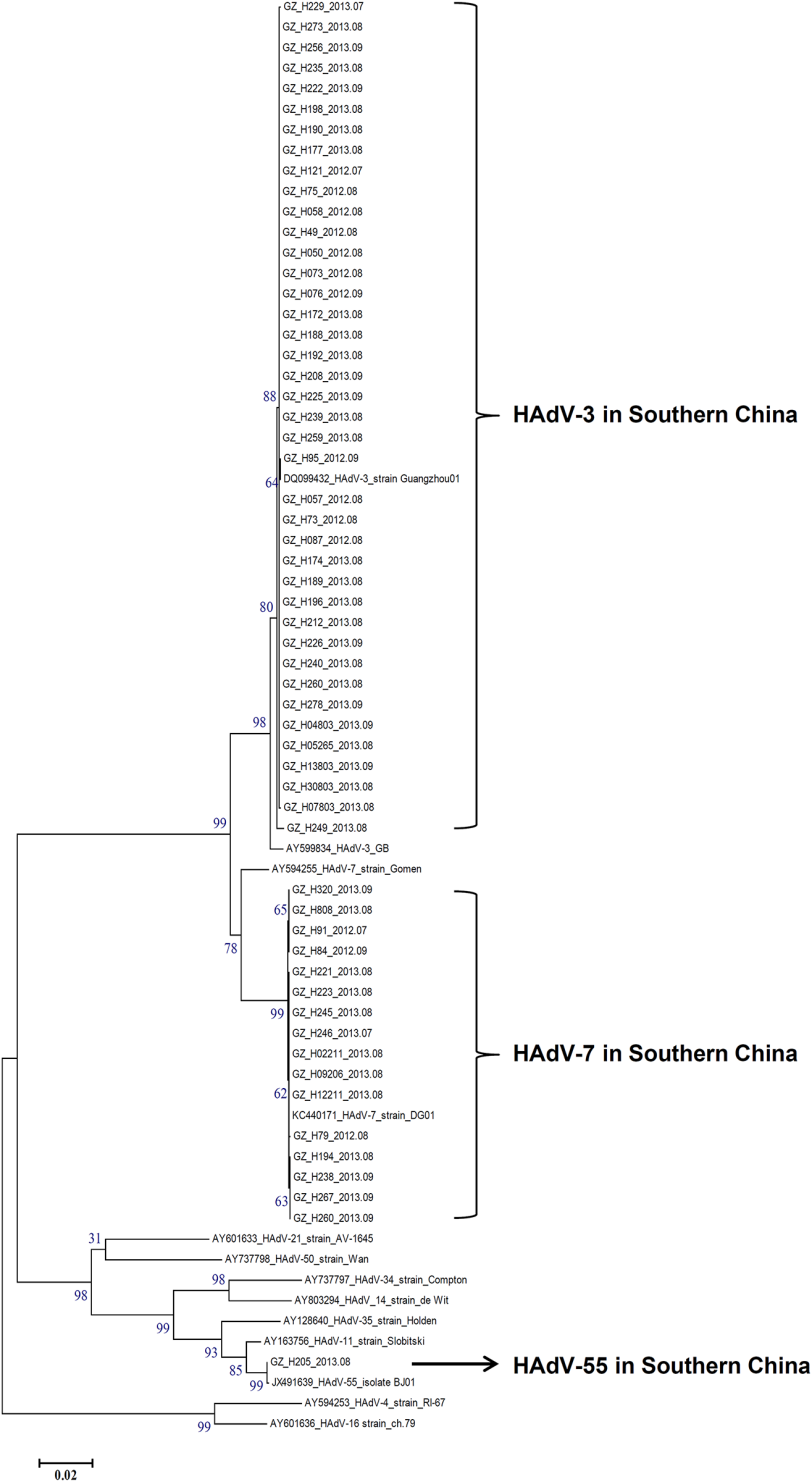
Phylogenetic analysis of seven HVRs of the hexon gene from HAdV-positive specimens from July through September, 2012–2013. Nucleotide sequences of the archived HVRs of HAdV-B reference strains were accessible from GenBank. The HVR sequences of two strains circulating in China were also included as reference: HAdV-3 strain Guangzhou01 (DQ099432) and HAdV-7 strain DG01 (KC440171). For reference, the year and month when the specimens were isolated were included in the strain names. Bootstrapped, neighbor-joining trees with 1,000 replicates were constructed using the MEGA 5.1.0 software (http://www.megasoftware.net) and by applying default parameters, with a maximum-composite-likelihood method. Bootstrap numbers shown at the nodes indicate the percentages of 1,000 replications producing the clade. A bootstrap value of 80 indicates robustness and confidence in the branching. The scale bar indicates units of nucleotide substitutions per site.

The HVR sequences of the hexon genes from these clinical strains isolated from hospitalized children have been deposited in GenBank under the accession numbers KR090744-KR090819. The phylogenetic tree was built based on the alignment of the nucleotide sequences of the seven HVRs with the sequences from relevant prototypes and circulating strains in China. Both the neighbor-joining and maximum likelihood trees provided the same HAdV clusters and similarly supported topology (Fig 3 and S1 Fig). This further confirmed the types of these isolates. HAdV-3 (n = 40) was the most prevalent during summer of 2012–2013. These HVRs formed a subclade with another China HAdV-3 isolate Guangzhou01 circulating in 2004 [19], but they were distinguished from the HAdV-3 prototype strain GB, all of which formed HAdV-3 clade. HAdV-7 was the second prevalent strain during the summers of 2012–2013 (n = 16). All the HVRs formed a subclade with another recently re-emergent HAdV-7 strain DG01 in 2011[24], indicating the hexon genes of HAdV-7 circulating in China were much conserved. However, they were discriminated from HAdV-7 prototype Gomen. The HVR sequence of HAdV-55 was very similar with the recently identified HAdV-55 strain BJ01 (Beijing; 2012) (bootstrap value 99) [15].

## Discussion

HAdV is one of the major causative pathogens of severe acute respiratory disease in human beings. Although most of respiratory infections caused by HAdVs are self-limiting, fatal infections also occur in children and adults [19, 25–27]. In this retrospective study, HAdV was the third most frequent pathogen which causes 12% children hospitalized due to ARI (495/4,130). Surprisingly, more than 6.5% of HAdV-positive patients with ARI needed intensive care, and 4.8% needed respiratory support with mechanical ventilation. Four children died of pneumonia due to HAdV infection, three of which had only HAdV infection. Thus, pediatrician must take HAdV infection into account when they have pediatric patients with ARI.

The previously reported HAdV infection rates among pediatric patients varied from 4.5% to 25% in different countries [18, 28–31]. For example, the HAdV infection rate in North-East Brazil is 25% [28], 17.9% in Italy [30]. In Northern China, the HAdV infections rate was 10.4%–20.1% [9, 31], which is consistent with our result in Guangzhou, Southern China (12.0%). However, the type distributions of HAdV in Southern and Northern China are completely different. In Southern China, most of HAdV-positive cases were caused by HAdV-3 (70% in this study) [6], followed by HAdV-7 (28%), while HAdV-7 dominated in Northern China (46.2%) [9]. However, another recent report found that HAdV-3 was the most predominant type in inpatient and outpatient children of Beijing, Northern China in 2003–2012, except for 2012, 2003 and 2007; HAdV-7 was the most predominant in 2012, and HAdV-3 and HAdV-7 co-circulated in 2003 and 2007 [32]. In their report, the molecular types were determined by nest PCR rather than sequencing; both outpatient and inpatient children were included, not just hospitalized children. These factors may account for the type distribution difference between the two reports in Northern China.

HAdV-55 was re-emergent in 2006 in China and named as type 55 in 2009 [33–34]. Subsequently it was re-emergent in Beijing in 2012 [35–36]. In this study, HAdV-55 was identified and reported for the first time in Guangzhou, Southern China in August 2013. Given that the high morbidity of severe pneumonia caused by HAdV-55, continuous monitoring of HAdVs types in children and adults are critically necessary. In this study, all the patients were inpatients and only HAdVs from the cases in seasonal peaks were molecular typed. These factors may account for only three types identified in this study.

A previous epidemiological study of HAdV infection in Guangzhou, China showed a tendency of gradual increase in infection rate in children, from 5% in 2006–2008 [29] to 9% in 2009–2012 [18]. Our study also indicates that HAdV infection in children increased to 9% (172/1867) in 2012 and 14% (323/2263) in 2013. For the pediatricians, more attention should be paid to this increasing HAdV infection rate during the past 7 years.

This study also revealed the incidence and pattern of seasonal prevalence of HAdV associated with hospitalized children in Southern China. Although HAdV infection can occur during the whole year, the peak season for HAdV infection in this study was summer, which was different from the previous reports in Northern China and other countries or regions. For examples, in Northern China, Mexico and Taiwan, the peak seasons were in winter and spring [9] [37] [38]. However, in Tennessee, USA [39] and Brazil [28], HAdV infection was observed all the year without a clear seasonality. The difference of seasonal distribution between Southern and Northern China remains unknown. Our hypothesis is that HAdV-3 predominant in Southern China might be more resistant to higher temperature than HAdV-7, which probably leads to more HAdV-3 cases in Southern China in summer. More experiments to compare the biological characteristics difference between HAdV-3 and HAdV-7 may confirm this hypothesis.

The co-infections with HAdV were also investigated in this study. In recent US study, multiple pathogens were detected in 26% of the hospitalized children [39]. In our study, 17.0% (84/495) of the HAdV positive children were co-infected with other pathogens detected from their nasopharyngeal swabs. Interesting, the recent HAdV epidemiology study showed that there was very high co-infection rate between HAdV and other pathogens (90.4%) in Northern China [9]. In our 495 HAdV-positive children, the most common co-infection pathogens with HAdV were MP and HBoV (48/84, 57.1% and 14/84, 16.7%, respectively), which was slightly different from that in Northern China [9], where RSV, RHV and HBoV were the most common co-infection pathogens with HAdV. The association between co-infection and severe illness is still unknown. The co-infection pathogens in the hosts might interact indirectly or directly with each other, but the interaction mechanism is poorly understood. Given the large proportion and diversity of the co-infection pathogens, further study is needed.

However, this study has some limitations. First, considering vast number of studied samples and the relatively limited resources, we performed the sequence analysis and molecular typing only from those samples in seasonal peaks, but not from the whole year. Therefore, the molecular typing of all HAdV positive samples in this study is not available. The cases in seasonal peaks might not reflect the difference in clinical manifestations and laboratory findings between the HAdV-3 and HAdV-7 in the whole year. Second, the sensitivity of current real-time fluorescent PCR kit for some pathogens (e.g., rhinovirus) is not optimal. It could not identify all the subtypes of rhinovirus, which might lead to the underestimation of rhinovirus infection rate.

In conclusion, this study reported the epidemiology among the HAdV-infected pediatric hospitalized patients with ARI in Guangzhou, Southern China in 2012–2013. HAdV was detected in 495 out of 4,130 (12.0%) children with ARI. The most prevalent HAdV type was HAdV-3 (70%) and HAdV-7 (28%) in summer. HAdV-55 was reported for the first time in Guangzhou in this study. Seasonal peaks of HAdV infections in Guangzhou occurred in summer, not in winter or spring. Co-infections with other respiratory viruses were common (17%). The clinical observations indicate that HAdVs is a major agent of ARI and HAdV infections can lead to severe clinical consequences, including death. Additional studies are needed to determine the role of HAdV infection in the severity of disease and their co-infections with other respiratory pathogens. More attention should be paid to the difference in circulating types and seasonal peaks of HAdVs between Southern and Northern China. Therefore, extensive and continuous surveillance for HAdV molecular epidemiology is necessary and HAdV vaccine should be developed to prevent the infection in children.

## Supporting Information

S1 Fig Phylogenetic analysis of seven HVRs of the hexon gene from HAdV-positive specimens from July through September, 2012–2013. The maximum-likelihood trees were constructed using the MEGA 5.1.0 software (http://www.megasoftware.net) and by applying default parameters.
(PDF)

S1 Table The incidence of HAdV infections in hospitalized children in 2012–2013. (XLSX)
