# Supplementary figures and images for: Molecular Identification and Epidemiological Features of Human Adenoviruses Associated with Acute Respiratory Infections in Hospitalized Children in Southern China, 2012-2013
Source: PLoS One. 2016 May 12;11(5):e0155412. doi: 10.1371/journal.pone.0155412 (PMC4865050; doi:10.1371/journal.pone.0155412)

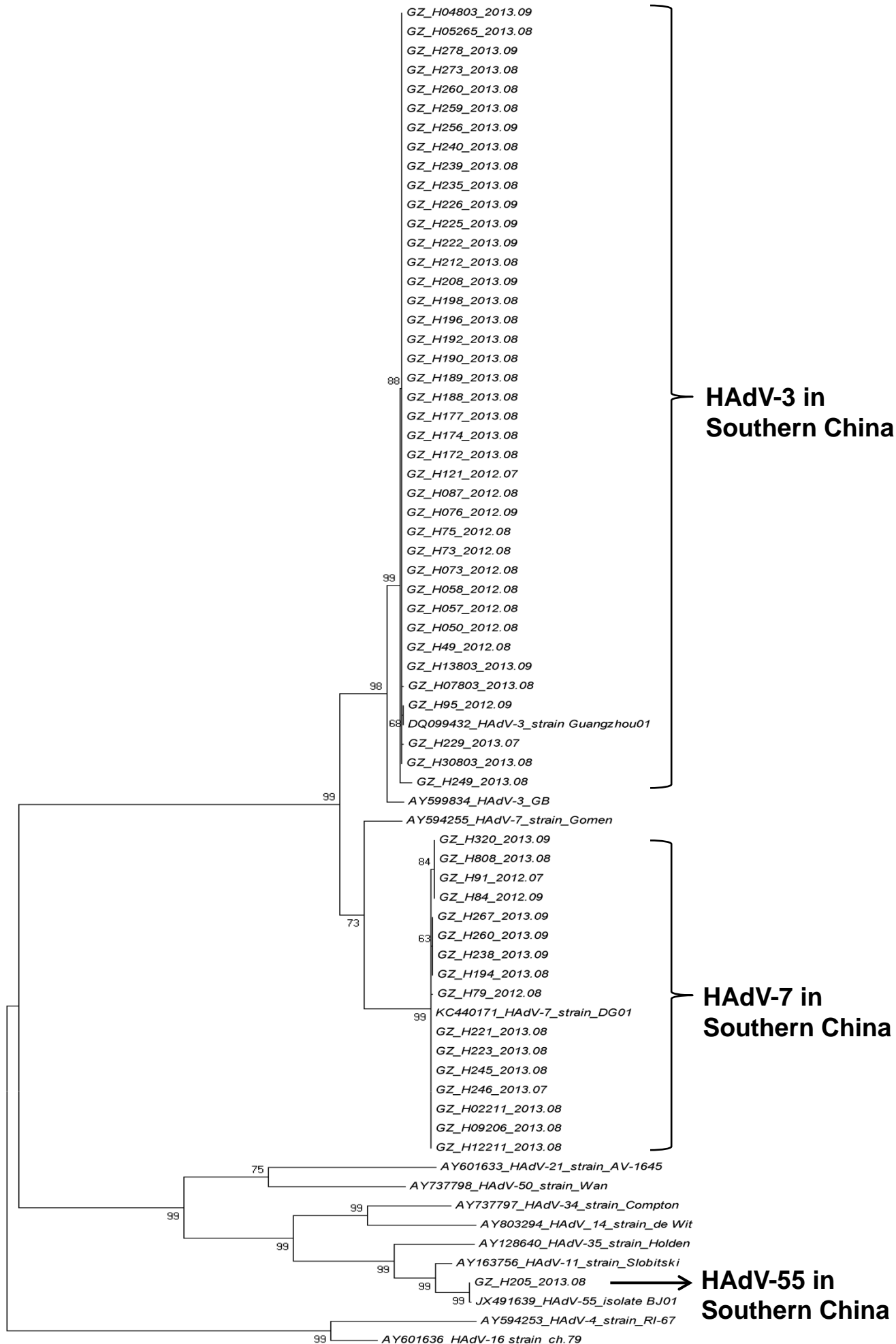

Supplement: S1 Fig — The maximum-likelihood trees were constructed using the MEGA 5.1.0 software (http://www.megasoftware.net) and by applying default parameters. (PDF) [file pone.0155412.s001.pdf]
